# Selection and Characterization of Bacteriocinogenic Lactic Acid Bacteria from the Intestine of Gilthead Seabream (*Sparus aurata*) and Whiting Fish (*Merlangius merlangus*): Promising Strains for Aquaculture Probiotic and Food Bio-Preservation

**DOI:** 10.3390/life13091833

**Published:** 2023-08-30

**Authors:** Sarah Cheriet, Sana Lengliz, Amel Romdhani, Paul Hynds, Mohamed Salah Abbassi, Taoufik Ghrairi

**Affiliations:** 1 Institute of Veterinary Research of Tunisia, University of Tunis El Manar, Tunis 1006, Tunisia; sarahcheriet5@gmail.com (S.C.); lengliz_sana@yahoo.fr (S.L.); romdhaniamel@live.com (A.R.); 2 Laboratory of Neurophysiology Cellular Physiopathology and Biomolecule Valorisation LR18ES03, Faculty of Sciences of Tunis, University Tunis El Manar, Tunis 2092, Tunisia; taoufik_ghrairi@yahoo.fr; 3 Laboratory of Materials, Molecules and Application LR11ES22, Preparatory Institute for Scientific and Technical Studies, University of Carthage, Tunis 1054, Tunisia; 4 Environmental Sustainability and Health Institute (ESHI), Technological University Dublin, Grangegorman, Dublin 7, D07 H6K8 Dublin, Ireland; hyndsp@tcd.ie; 5 Research Laboratory «Antimicrobial Resistance» LR99ES09, Faculty of Medicine of Tunis, University of Tunis El Manar, Tunis 1006, Tunisia

**Keywords:** marine fish, lactic acid bacteria, bacteriocin, fish health, bio-preservation

## Abstract

This study sought to evaluate the probiotic properties and the food preservation ability of lactic acid bacteria isolates collected from the intestines of wild marine fishes (gilthead seabream (*Sparus aurata*) (*n* = 60) and whiting fish (*Merlangius merlangus*) (*n* = 40)) from the Mediterranean sea in the area of Mostaganem city, Algeria. Forty-two isolates were identified as: *Enterococcus durans* (*n* = 19), *Enterococcus faecium* (*n* = 15), *Enterococcus faecalis* (*n* = 4), *Lactococcus lactis* subp. *lactis* (*n* = 3), and *Lactobacillus plantarum* (*n* = 1). All isolates showed inhibition to at least one indicator strain, especially against *Listeria monocytogenes*, *Staphylococcus aureus*, *Paenibacillus larvae*, *Vibrio alginolyticus*, *Enterococcus faecalis*, *Bacillus cereus*, and *Bacillus subtilis*. In all collected isolates, PCR analysis of enterocin-encoding genes showed the following genes: *entP* (*n* = 21), *ent*1071A/B (*n* = 11), *entB* (*n* = 8), *ent*L50A/B (*n* = 7), *ent*AS48 (*n* = 5), and *entX* (*n* = 1). Interestingly, 15 isolates harbored more than one *ent* gene. Antimicrobial susceptibility, phenotypic virulence, and genes encoding virulence factors were investigated by PCR. Resistance to tetracycline (*n* = 8: *tetL* + *tetK)*, erythromycin (*n* = 7: 5 *ermA*, 2 *msrA*, and 1 *mef*(A/E)), ciprofloxacin (*n* = 1), gentamicin (*n* = 1: *aac*(6′)-*aph*(2″)), and linezolid (*n* = 1) were observed. Three isolates were gelatinase producers and eight were α-hemolytic. Three *E. durans* and one *E. faecium* harbored the *hyl* gene. Eight isolates showing safety properties (susceptible to clinically relevant antibiotics, free of genes encoding virulence factors) were tested to select probiotic candidates. They showed high tolerance to low pH and bile salt, hydrophobicity power, and co-culture ability. The eight isolates showed important phenotypic and genotypic traits enabling them to be promising probiotic candidates or food bio-conservers and starter cultures.

## 1. Introduction

Lactic acid bacteria (LAB) are cocci- or rod-shaped Gram-positive bacteria, not mobile, nonsporulating, anerobic or facultatively aerobic, and producing lactic acid as the major fermentation product from the carbohydrate metabolism pathways [1]. LAB include more than 60 genera; however, the most important are: *Lactobacillus*, *Lactococcus*, *Leuconostoc*, *Pediococcus*, *Streptococcus*, *Aerococcus*, *Alloiococcus*, *Carnobacterium*, *Dolosigranulum*, *Enterococcus*, *Oenococcus*, *Tetragenococcus*, *Vagococcus*, and *Weissella*, with *Lactobacillus* being the largest genus, comprising over 261 species [2,3]. LAB are able to inhibit pathogens by producing antibacterial compounds, including bacteriocin, lactic acid, hydrogen peroxide, acetaldehyde, and diacetyl which inhibit pathogens’ activities [4]. The majority of LAB possess a number of crucial metabolism traits, including the ability to make acid and aroma, hydrolyze proteins, and produce viscous exopolysaccharides. In addition, LAB can tolerate acidic pH and bile salts, which enable them to survive in gut systems [5]. These bacteria can colonize the intestinal mucus, where they assist in the processing and uptake of feed, promoting the growth of the animals and humans, as well as stimulation of the specific and non-specific immune systems and gut immunity [6,7]. The vast majority of LAB have the status ‘Generally Recognized As Safe’ (GRAS) according to the U.S. Food and Drug Administration (FDA). All the aforementioned traits have enabled LAB to be used for several purposes. Indeed, LAB are used as starter cultures, playing an important role in fermentation processes, the bio-preservation of meat and meat products, as a probiotic for livestock and aquaculture, and to promote human health [5,8,9,10,11]. LAB probiotics have been largely used in livestock [12] and their application in aquaculture is growing as well [7]. 

According to FAO (2019) [13], over 158 million tons (89%) of total fishery and aquaculture production was used for direct human consumption, and 44% of the fish destined for human consumption was in live and fresh form. The response to the rapid increase in the human population and the need for inexpensive sources of protein as well as the decline in fish catches from inland natural lakes have all created the need to develop aquaculture rapidly [14,15]. Since aquaculture grew faster than any other food-producing sector, nowadays aquaculture is one of the most important industries to provide the animal protein for the human consumption worldwide [16]. However, globally, the growth of intensive aquaculture industries has enhanced the incidence of infectious disease (virus, parasites, bacteria) outbreaks in the farmed species, leading to massive mortalities and economic loss, which has been estimated as more than USD 6.0 billion per year worldwide [17,18]. Most of the bacterial disease outbreaks are caused by *Aeromonas*, *Pseudomonas*, *Citrobacter*, *Streptococcus*, *Edwardsiella*, *Proteus*, *Staphylococcus*, and *Vibrio* species [18,19]. Many approaches have been developed to control and mitigate against the effect of pathogens in aquaculture such as vaccines, immunostimulants, use of chemical additives (water disinfection), and antimicrobial compounds [20]. In addition, any alternatives have been developed to avoid antimicrobial and chemical additives use in aquaculture such as phytocompounds (plant extracts), prebiotics, bacteriophages, immunostimulants (including polysaccharides, hormones, vitamins, different components of bacteria, biologically active materials), and probiotics [9,21]. Some of the most studied probiotic candidates are LAB and *Bacillus*. Indigenous LAB strains (isolated from the intestinal tract) exhibiting antibacterial activity and safety traits are efficiently used in the aquaculture of various fish species, since they are well adapted to the fish microbiota and to aquatic environment [22].

Taken together, research on the development of new biologic strategies to fight pathogens (including antimicrobial resistant bacteria) and probiotic candidates either for livestock or for aquaculture is urgently needed. Therefore, the aim of this study was to evaluate the biotechnological and the probiotic properties of LAB isolates collected from the intestine of wild fish (seabream and whiting fish) collected from the Mediterranean Sea in the area of Mostaganem city, Algeria.

## 2. Materials and Methods

### 2.1. Isolation of LAB Strains

The samples of wild fish (gilthead seabream (*Sparus aurata*) (*n* = 60) and whiting fishes (*Merlangius merlangus*) (*n* = 40)) were collected from the Mediterranean Sea in the area of Mostaganem city, Algeria. Fish were collected, stored in ice, and then analyzed within 24 h after catching. Fish were washed with sterile distilled water and dissected to open the gastrointestinal tract under laminar airflow conditions. The gastrointestinal tract of each sample was homogenized using de Man, Rogosa and Sharpe (MRS) broth (BD Difco™, Strasbourg, France), incubated for 4 h at 37 °C, and then centrifuged at 5000× *g* for 5 min. After centrifugation, the supernatant was serially diluted and spread-plated on MRS agar (BD Difco™, Strasbourg, France) plates and incubated at 30 °C for 24–48 h. When growth is observed, from each positive sample, one to three colonies with typical LAB morphology were randomly selected and further identified by conventional bacteriological tests (Gram staining, catalase test, oxidase test). Among those isolates, only one presenting the characteristics of LAB was maintained for further characterization. Collected isolates were identified by API 50 CHL system and Api20 Strep kit (Biomérieux, Marcy l’Etoile, France) according to the supplier instructions and confirmed by PCR experiment as reported previously [23].

### 2.2. Antibacterial Activity Testing 

For the determination of antibacterial activity, we proceeded as described by Jaouani et al. [24]. *Listeria monocytogenes* ATCC 43256, methicillin-resistant *Staphylococcus aureus* [25], *Escherichia coli* ATCC 25922, *Enterococcus faecalis* JH2-2, *Vibrio alginolyticus* [26], *Salmonella typhimurium* 4,5,12:i:- (monophasic) LSP 389/97, *Pseudomonas aeruginosa* ATCC 27853, *Bacillus cereus* [24,27], *Bacillus subtilis* [24,27], and *Paenibacillus larvae* [24,28] were used as indicator strains. A 100 μL of an overnight culture of the indicator strain was added to 20 mL brain heart infusion (BHI) broth (Oxoid, Milan, Italy) supplemented with 0.7% agar, mixed and poured onto Petri dish. A colony of the isolate to be tested for antimicrobial activity was transferred with a sterile toothpick to the soft agar seeded with the indicator bacteria. Plates were incubated for 24 h at 37 °C in aerobic conditions. Five to eight of the collected isolates can be tested per one Petri dish. The antimicrobial activity was visually detected by clear inhibition zones around the tested strain and scores were assigned based on the diameter of the inhibition halo: ++++ = clear zone > 15 mm, +++ = clear zone 10–15 mm, ++ = 5–9.9 mm, + = clear zone 1–4.99 mm, – = no zone [24]. This test was performed in triplicate.

### 2.3. Nature of Inhibition Substances 

Tested isolates were grown overnight at 37 °C in 10 mL of MRS broth (BD Difco™, Strasbourg, France). Supernatants were collected by centrifugation at 4 °C during 10 min at 10,000× *g* and sterilized by filtration using 0.22 μm filters. Their antibacterial activity was tested by the well diffusion assay as follows: 150 µL aliquot of an overnight culture of the indicator bacteria was mixed with 15 mL of BHI broth (Oxoid, Milan, Italy) supplemented with 0.8% agar and poured into plates. Wells were punched in the agar and filled with 100 µL of the supernatant extracts. Simultaneously, to test the proteinaceous nature of the inhibitory activity, sterilized supernatants were adjusted to pH 7 with 1 mol L^−1^ NaOH, to eliminate the effect due to organic acids, and 100 μL were treated with 5 μL of proteinase K (25 mg mL^−1^) (Sigma-Aldrich, Munich, Germany). Both neutralized and proteinase K-treated supernatants were added (100 µL) to wells and plates were incubated overnight at 37 °C. The production of bacteriocins or bacteriocin-like compounds by the tested strains was confirmed by the presence of inhibition zones around the well containing the pure and the neutralized culture broth and the absence of the halo around the well containing the proteinase K [29]. 

### 2.4. Resistance to Temperature Effect

To analyze thermal stability, supernatants were heated to 60 °C for 30 min, 80 °C for 10 min, 100 °C for 5 min, autoclaved at 121 °C for 15 min, and then cooled to room temperature before being tested for antibacterial activity as reported above [26]. The stability of inhibition activity was also tested at laboratory temperature (25 °C), at 4 °C, and at −20 °C for one and two weeks.

### 2.5. PCR Detection of Bacteriocin Genes

Genomic DNA was extracted from isolates grown overnight in 5 mL of BHI broth at 37 °C using the protocol previously described by Lengliz et al. [27]. The *entA*, *entB*, *entP*, *entQ*, *entL50A/B*, *entAS-48*, *ent31*, *entX*, and *ent1071A/B* genes encoding the most relevant enterocins were targeted by PCR using the primers and conditions as reported previously [27]. Amplicons were observed by electrophoresis in 2% agarose gels containing 0.5 µg L^−1^ ethidium bromide in 0.5 X TBE. The 100-bp ladder (Sigma-Aldrich Chemie, Steincheim, Germany) was used as molecular weight marker.

### 2.6. Antimicrobial Susceptibility Testing

The antibiotic susceptibility of all isolates was carried out by the agar disc diffusion method on Mueller–Hinton agar (Bio-Rad, Hercules, CA, USA) plates according to the recommendation of the Clinical and Laboratory Standards Institute guidelines [30]. The following antibiotics were employed (µg/disc): ampicillin (10 µg), erythromycin (15 µg), gentamicin (500 µg), streptomycin (300 µg), linezolid (30 µg), vancomycin (30 µg), tetracycline (30 µg), and teicoplanin (30 µg). 

### 2.7. Gelatinase Production 

Gelatinase production was evaluated using agar plates containing gelatin [31]. Plates containing Brain Heart Infusion agar (BHIA) supplemented with 30 g L^−1^ gelatin (Oxoid, Hampshire, UK) and 10 g L^−1^ peptone (Becton-Dickinson Corp., Cockeysville, MD, USA) were streaked by the bacteriocinogenic isolates (5 to 6 streaks by Petri dish) and incubated at 37 °C for 24 h. Then, the plate surface was covered with a saturated solution of ammonium sulphate (Sigma-Aldrich Chemie, Steincheim, Germany). Presence of a transparent zone around colonies indicated gelatinase activity [32]. Previously characterized enterococci strains of our collection [27,33] were used as positive controls. 

### 2.8. DNase Production

For detection of DNase, one colony from a 18 h old culture from each tested isolate was streaked on DNase agar medium (Bio-Rad, Hercules, CA, USA) (5 to 6 streaks by Petri dish). After incubation for 24 h at 37 °C, plates were flooded with 3% HCl for 5–10 min and observed for the presence of a clear zone around colonies. *S. aureus* ATCC 25923 was used as the positive control [27].

### 2.9. Hemolytic Activity 

The hemolytic activity was determined by streaking a single colony on Columbia agar supplemented with 5% (*v*/*v*) sheep blood. After incubating for 24 h at 37 °C, hemolysis was classified as α-hemolysis (green zone around colonies), β-hemolysis (clear zone around colonies), and γ-hemolysis (no halo around colonies) [34]. *E. faecalis* ATCC 29212 was used as the β-hemolytic strain.

### 2.10. Genetic Screening for Virulence Potential and Detection of Genes Encoding Antimicrobial Resistance

Isolates were tested for ten virulence genes commonly reported in *Enterococcus* spp. and other LAB: *hyl* (hyaluronidase), *esp* (enterococcal surface protein), *geIE* (gelatinase), *agg* (aggregation substance), *ace* (collagen adhesion), *efA_fs_* (cell wall adhesion), *CylL_L/s_* (cytolysin), *cob* (sex pheromones), *cpd* (sex pheromones), and *ccf* (sex pheromones) [27]. For antimicrobial resistant isolates, genes encoding a corresponding resistance marker were detected by PCR: gentamicin/kanamycin/tobramycin (*aac*(6’)-Ia-*aph*(2”)-Ie); streptomycin (*ant*(6)-Ia), erythromycin (*erm*(B), *erm*(A), *erm* (C), *msr*(A), and *mef*(A/E)); tetracycline (*tet*(M), *tet*(L), *tet*(O), *tet*(K), and *tet*(S)); vancomycin/teicoplanin (*van*A, *van*B); and linezolid (*optr*A), as reported previously [35,36,37,38]. Positive controls from previously characterized *S. aureus* and enterococci strains [25,35,36,38,39,40,41,42,43] were used in all PCR experiments. 

### 2.11. Assessment of the Probiotic Potential of Safety Isolates 

Bacteriocinogenic isolates evaluated as safe according to the absence of acquired antimicrobial resistance markers, free of virulence genes and phenotypic virulence traits were selected to assess the probiotic potential of these isolates by studying the following.

#### 2.11.1. Growth at Different pH Values 

To test the effect of pH on all probiotic candidates’ strains, the used method was as described by Lengliz et al. [27] using microtiter plates and MRS broth with pH 4, 5, 6, 7, and 8. Optical density readings of bacteria growth were recorded every hour for 28 h using a spectrophotometer (OD_620_) (Labsystems multiscan RC). Cultures grown in MRS broth served as the control (pH 6.5).

#### 2.11.2. Gastric and Bile Tolerance 

The evaluation of the resistance to gastric acidity was carried out according to the method described by Argyri et al. [44]. Isolates were harvested by centrifugation (10,000× *g*, 10 min, 4 °C), washed twice with a sterile PBS solution (pH 7.3), then resuspended in 1 mL of PBS, and diluted (1:100) in a PBS solution adjusted to pH 2.5. Resistance was assessed in terms of the number of viable colonies and listed in duplicate on Bile Esculin Agar (BEA) (Bio-Rad, Hercules, CA, USA) after incubation at 37 °C for 0; 0.5; 1; 2; and 3 h, which reflects the time spent by food in the stomach. Bile tolerance was determined by streaking single colonies on TH agar plates containing 1% (*w*/*v*) oxgall bile (Sigma-Aldrich Chemie, Steincheim, Germany). Plates were incubated at 37 °C for 24 h and visually examined for the growth.

#### 2.11.3. Hydrophobicity

Hydrophobicity of the selected isolates was determined using Congo Red staining as reported by Leyva-Madrigalet et al. [45]. One colony of each isolate was streaked on MRS agar plates containing 2% of NaCl and 0.03% Congo Red (Sigma-Aldrich Chemie, Steincheim, Germany) and incubated at 37 °C for 24–48 h. Red colonies were considered hydrophobic and white or colorless colonies were considered non-hydrophobic [46].

#### 2.11.4. Coexistence of Isolates

The coexistence between the safety isolates was examined by a cross-streak method [47]. The antagonism against each other was visualized by the observation of an inhibition zone at the angle of intersection between the first isolate and the remaining ones.

## 3. Results and Discussion

### 3.1. Selection and Identification of Bacteriocinogenic LAB Isolates

From the two fish species, 42 LAB isolates were collected. The majority of isolates belonged to *Enterococcus* genera (*n* = 38) (Supplementary Materials, Figures S1–S3), and the remaining were identified as *Lactococcus lactis* subp. *lactis* (*n* = 3) and *Lactobacillus plantarum* (*n* = 1). LAB have been commonly isolated from the intestinal tract of fish with a predominance of *Carnobacterium*, *Enterococcus*, *Lactobacillus*, *Lactiplantibacillus*, *Vagococcus*, *Lactococcus*, and *Weissella* genera [48,49,50,51]. Similar to our findings, Chahad et al. [48] have reported the predominance of enterococci isolates from farmed marine fish (gilthead seabream and European seabass). Overall, the collected bacteria species have high adaptability to various environments, including dairy and plant products [52] and marine and freshwater fish [53,54]. The enterococci isolates were predominately presented by the *E. durans* (19 isolates: 10 *S. aurata* + 9 *M. merlangus*) and *E. faecium* (15 isolates: 9 *S. aurata* + 6 *M. merlangus*) species and only four isolates were identified as *E. faecalis* (2 *S. aurata* + 2 *M. merlangus*). In human clinical settings, *E. faecalis* and *E. faecium* are the major enterococcal species implicated in human diseases, including bacteremia, urinary tract infections, and wound infections; however, other species such as *E. durans*, *E. casseliflavus*, *E. gallinarum*, and *E. mundii* are rarely isolated [55]. *Enterococcus seriolicida* has been for a short time considered as a fish pathogen [56]; however, advanced molecular methods showed that *E. seriolicida* is a junior synonym of *Lactococcus garvieae*, a causative agent of septicemia and meningoencephalitis in freshwater and salt water fish [57]. 

*L. plantarum* belong to the *Lactobacillus* genus which is the main and most diverse LAB group. *L. plantarum* species is found in wide ecological niches such as dairy products, vegetables, meat, silage, wine, gastrointestinal, vaginal, and urogenital tracts [58]. This ubiquity of *L. plantarum* permits amazing capabilities of adaptation and metabolic pathway diversities [58,59]. The health claims of *L. plantarum* permits its development in different probiotic formulations, and its antibacterial properties are interesting for food safety as in biopreservation technology. Indeed, *L. plantarum* is used in the fermentation of dairy products, fermented meat products, fermented vegetables, and beverages [58,59]. Similarly, *Lactococcus lactis* subp. *lactis* plays an important role in the dairy industry as a common part of many fermented products and as a crucial part of starter cultures [60,61]. 

All isolates showed inhibition to at least one indicator strain, and only *E. coli* and *P. aeruginosa* strains were not inhibited. Indeed, *L*. *monocytogenes*, methicillin resistant *S. aureus* (MRSA), *P. larvae*, *Vibrio alginolyticus*, *E. faecalis*, *B. cereus*, and *B. subtilis*, were inhibited by 39, 37, 37, 16, 10, and 8 isolates, respectively. It is well known that LAB exhibit antibacterial activities against Gram-positive bacteria such as *L. monocytogenes*, *S. aureus*, *E. faecalis*, *B. cereus*, and *B. subtilis* [24,26,62]. Food was identified as the first source of infection of *L. monocytogenes* in humans, and today it is a public health concern related to septicemia, meningitis, gastroenteritis, pneumonia, and abortion [63]. According to the European Food Safety Authority (EFSA) data in 2016 [64], *L. monocytogenes* are prevalent in fish and fishery products; therefore, fish can be a source of disease transmission to humans [65,66]. In addition, the bacteria can survive in relatively low water conditions/activity, resistance to salt, and freezing temperatures. Therefore, the anti-*Listeria* activity exhibited by a high number of our isolates is an interesting trait indicating possible use of such isolates in the bioconservation of fish products and might also imply the possibility of in vivo exclusion of *L. monocytogenes* in the intestine of the two studied fish species or others. Although *S. aureus* has not been implicated in fish diseases, the presence of bacteria is considered a contamination before or after harvest probably by fish handlers colonized by *S. aureus* [67]. Enterotoxigenic *S. aureus* can cause gastroenteritis via humans eating contaminated fish and its products; however, skin infections are caused by TSST-1 toxin-producers isolates leading to toxic shock syndrome [68]. Similarly, *B. cereus* is one of the leading etiological agents of toxin-induced foodborne diseases. It is estimated that 1.4–12% of foodborne outbreaks worldwide can be attributed to *B. cereus* [69]. Therefore, inhibition of *S. aureus* and *B. cereus* is of great interest to fight foodborne diseases caused by these pathogens. On the other hand, one of the interesting findings in our study is the inhibition of *V. alginolyticus* by 37/42 isolates. Indeed, *Vibrio* species, a Gram-negative bacteria, cause vibriosis in animals, including fish, and humans posing a risk of zoonotic disease in aquaculture professionals and consumers of aquatic products. *V. alginolyticus*, *Vibrio anguillarum*, *Vibrio campbellii*, *Vibrio harvey*, *Vibrio vulnificus*, and *Vibrio parahaemolyticus* are the most important species found in infected fish [70]; However, *V. cholerae*, *V. alginolyticus*, *V. vulnificus*, *Vibrio damselae*, *Vibrio hollisae*, *Vibrio metschnikovi*, and *V. parahaemolyticus* species infect humans. Thus, the high inhibition power of the 37 isolates against *V. alginolyticus* can be valorized in aquaculture (probiotic application) or in the bioconservation of seafood items and fish products. Several other studies have reported high inhibitory power of LAB and *Enterococcus* spp. of fish origin as well as other sources against fish-associated *Vibrio* species [7,71,72]. Furthermore, 37 isolates were able to inhibit *P. larvae*, the causative agent of American foulbrood (AFB) disease, which is by far the most virulent and deleterious bacterial disease that causes fatal foulbrood infections in honey bees (*Apis mellifera*) [73]. Unfortunately, no cure exists for this notorious disease; thus, for the hive (as well as the potentially contaminated equipment) incineration is commonly used to prevent the spread of AFB in clinically diseased colonies. Antibiotic use for AFB control in beekeeping practice is associated with a growing awareness of the emergence of resistant *P. larvae* strains, honey bee immunological deficiencies, and the dissemination of antibiotic resistance genes, disturbed honey bee microbiota, and reduced lifespan of honey bees [73]. Therefore, these strains exhibiting anti-*P. larvae* present potential candidates to be used as probiotics to combat this virulently contagious disease [24,74].

### 3.2. Properties of Antibacterial Substances and Molecular Identification of Bacteriocins

LAB produce a range of metabolites with antimicrobial action, which include hydrogen peroxide, lactic acid, acetic acid, and low molecular weight substances (diacetyl, fatty acids, reuterin, reutericyclin), antifungal compounds (phenyl lactate, propionate, hydroxyphenyl lactate), and bacteriocins [10]. Therefore, to differentiate between bacteriocins from other metabolites, proteinase K was used to treat the neutralized culture supernatants of bacteriocinogenic isolates. The inhibitory activity was lost for all tested isolates, indicating a proteinaceous nature of the inhibitor substances. Furthermore, results of the sensitivity tests to heat treatment showed that the bioactive substances were thermostable at 60 °C, at 80 °C, and at 100 °C; however, this activity was clearly lost after autoclaving (121 °C for 15 min). Storage at different conditions showed activity after 2 weeks storage at room temperature and at 4 °C (fridge temperature). However, it is interesting to note that since we evaluated these activities by visual observation we noted a 2 to 3 mm reduction in the inhibitory zone around wells in comparison to untreated substances. The most stable activity of stored substances was observed at −20 °C. The high stability of the inhibitor substances makes them a robust agent that can be used in the food industry to keep out spoilage microbes [75].

The well-known genes encoding bacteriocin in enterococci were investigated by PCR. Among the 42 bacteriocinogenic isolates, 11 were free of all investigated genes. These isolates belonged to *E. faecium* (*n* = 1), *E. faecalis* (*n* = 1), *E. durans* (*n* = 6), *L. lactis* subp. *lactis* (*n* = 2), and *L. plantarum* (*n* = 1). The *entP* gene was the most common (*n* = 21) followed by *ent*1071A/B (*n* = 11), *entB* (*n* = 8), *ent*L50A/B (*n* = 7), *ent*AS48 (*n* = 5), and *entX* (*n* = 1). Interestingly, 15 isolates harbored more than one *ent* gene: *ent*AS48 + *ent*1071A/B + *ent*B + *ent*L50A/B (*n* = 1), *ent*P + *ent*B + *ent*1071A/B + *ent*L50A/B (*n* = 1), *ent*P + *ent*B + *ent*1071A/B (*n* = 1), *ent*P + *ent*B + *ent*L50A/B (*n* = 1), *ent*B + *ent*1071A/B + *ent*L50A/B (*n* = 2), *ent*P + *ent*1071A/B (*n* = 2), *ent*P + *ent*L50A/B (*n* = 1), *ent*P + *ent*AS48 (*n* = 1), *ent*P + *ent*X (*n* = 1), *ent*P + *ent*B (*n* = 1), *ent*B + *ent*L50A/B (*n* = 1), and *ent*AS48 + *ent*1071A/B (*n* = 2) (Table 1). The *ent*P, *ent*A, and *ent*B genes are commonly reported in bacteriocinogenic enterococci isolates from various origins and are frequently associated to each other or to other *ent* genes [24,27,71,76]; however, *ent*1071A/B, *ent*L50A/B, *ent*AS48, and *entX* are sporadically reported [77,78]. The bacteriocinogenic- but *ent*-free isolates might harbor other genes not investigated in our study [77] or yet unknown bacteriocin genes. *L. lactis* subp. *lactis* and *L. plantarum* mainly harbor non-enterocin genes, such as curvacin A, plantaricin A, sakacin P, and nisin [79] which might explain the absence of investigated enterocin genes in the two out of the three *L. lactis* subp. *Lactis* and the *L. plantarum* isolate. 

### 3.3. Safety Assessments and Probiotic Properties of Bacteriogenic LAB Isolates

For the safe application of probiotics and starter candidates and anti-spoilage microbes, special attention needs to be given to the presence of possible genetic determinants of virulence factors. In addition, it is important to investigate the presence of virulence factors by both molecular and phenotypic procedures. All our isolates were unable to degrade DNA; however, three produced gelatinase and eight were α-hemolytic. DNA degradation, gelatinase production, and lysis of red blood cells (erythrocytes) (principally β-hemolysis) are considered as important virulence factors in enterococci and other bacteria [55]. The *gelE* gene encodes for an extracellular zinc endopeptidase hydrolyzing gelatin, collagen, hemoglobin, and other bioactive compounds [80], and was absent in the three gelatinase producer isolates. Until now, only *gelE* gene is known to encode gelatinase production in enterococci, other unknown gene(s) might be harbored by these isolates. Moreover, false negative PCR results are possible, especially when the sequences are not known and the alleles are likely divergent among strains. Among the other investigated genes, only the *hyl* gene, which encodes a hyaluronidase enzyme, was detected in three *E. durans* and one *E. faecium* isolate. The *hyl* gene has been scarcely detected in different enterococci species; however, it seems characteristic to vancomycin-resistant *E. faecium*. *hyl* and *esp* (encoding enterococcal surface protein) genes and the insertion sequence IS*16* as well as resistance to ampicillin has identified *E. faecium* strains of clinical origin [81]. In our case, the *hyl*-positive *E. faecium* isolate was ampicillin susceptible and *esp*-free.

The occurrence of acquired antimicrobial resistance phenotypes and genotypes in bacteriocinogenic LAB investigated as potential bioconservers or as probiotic/starter candidates is also considered as a hazard. Genes encoding antimicrobial resistance may be transferred through conjugative mobile elements (integrons, transposons, and plasmids) to other resident microbiota including pathogenic ones in the host gut of treated animals or to the gastrointestinal tract of consumers as well as to the aquatic environment [82]. In our isolates, 30 were susceptible to all tested antibiotics. Few isolates were resistant to tetracycline (*n* = 8), erythromycin (*n* = 7), ciprofloxacin (*n* = 1), gentamicin (*n* = 1), and to linezolid (*n* = 1). The aforementioned resistance markers have been previously reported in several LAB including enterococci isolates [27,83]. Some LAB are intrinsically resistant to some antimicrobials; however, the presence of intrinsic resistance genes is undesirable but may not constitute a safety issue, since these genes are not easily transmitted to other pathogens and LAB are very rarely involved in infections. The most important for safety assessment is, therefore, the occurrence of transferable antimicrobial resistance genes such as genes encoding resistance to aminoglycosides, macrolides-lincosamides-streptogramins (MLS), glycopeptides, tetracyclines, and linezolid which are mainly plasmid-borne. The eight tetracycline resistant isolates harbored both *tetL* and *tetK* genes. For the seven erythromycin-resistant isolates, each of *ermA*, *msrA*, and *mef*(A/E) genes were detected in five, two, and one isolates, respectively, and one of them harbored both *ermA* and *msrA* genes. Resistance to gentamicin was encoded by the *aac*(6′)-*aph*(2″) in the resistant isolate. However, *optrA* and *ant*(6)-Ia genes encoding linezolid and high-level streptomycin resistance were not detected. Those resistance phenotypes and genotypes are commonly detected in enterococci isolates and several other LAB species [5,27].

Among our studied isolates, L. lactis subsp. lactis S40, *E. durans* S4, *E. durans* S50, *E. durans* S32, *E. faecium* S6, *E. faecium* S7, *E. faecium* S10, and *E. faecium* S21 isolates were selected according to their high inhibition spectrum, antibiotic susceptibility, and absence of relevant virulence factors /genes in order to evaluate their potential use as probiotic candidates. Acid and bile salt resistance are important criteria for selecting probiotic LAB, and are some of the main factors affecting the survival probability of LAB in the stomach and digestive tract and eliciting positive effects in the host [84]. The probiotic LAB must survive in an acidic stomach environment in order to reach the small intestine, and then to resist the deleterious effects of bile salts in order to survive in gastrointestinal transit and to colonize the gut. The eight isolates survived at pH5 and pH6; however, at pH4 no growth was observed. In addition, the eight isolates survived efficiently without important loss in the cell viability in MRS at pH2.5 during 3 h indicating tolerance to gastric conditions. Similarly, all isolates were able to grow in 1% bile salts. These findings are not surprising, indeed several studies, with few exceptions, reported that LAB can survive under gastric conditions (low pH) and resist bile salts; moreover, these traits are particularly observed in LAB species of human and animal microbiota [27,33,62]. Another important criterion to be considered when selecting potential probiotic candidates is the strain surface hydrophobicity; indeed, high hydrophobicity of LAB strains correlate with their attachment to intestinal mucosal and epithelial cells [8]. The used test of hydrophobicity showed that our selected isolates had hydrophobic structures in the cell wall, enabling them to colonize efficiently in the intestine. The test of coexistence was realized to assess the possibility of using multi-strains as probiotics. No single isolate was able to inhibit other strains; thus, all were compatible in the coexistence assay. 

## 4. Conclusions

The intestine of wild fish (gilthead seabream and whiting fish) contained LAB with high antibacterial activity against human and animal pathogenic bacteria and the fish pathogen *V. alginolyticus*. The safety assessment and in vitro evaluation of probiotic traits identified eight strains (*L. lactis* subsp. *lactis* S40, *E. durans* S4, *E. durans* S50, *E. durans* S32, *E. faecium* S6, *E. faecium* S7, *E. faecium* S10, and *E. faecium* S21) presenting promising probiotic candidates that can be used as single-strain or multi-strain especially in aquaculture. These eight strains also showed important biotechnological traits enabling them to be used as food bio-conservers and starter culture. Surely, further in vitro and in vivo investigations as well as genomic studies by whole genome sequencing analysis will provide valuable insights into the efficacy of those isolates. Indeed, the WGS analysis of *L. lactis* subsp. *lactis* S40 is under investigation and results will be published in the very near future.

## Figures and Tables

**Table 1:** Phenotypic and genotypic characteristics of the 42 LAB isolates collected from gilthead seabream (*Sparus aurata*) and whiting fish (*Merlangius merlangus*).

Isolates	Origin	Spectrum of Inhibition *									*ent* Genes	Virulence Genes	Resistance Profile/ Resistance Gene	DNase/Gelatinase/ Hemolyze
		B.s	B.c	L.m	S.a	E.f	P.l	E.c	P.a	V.a				
*L. lactis* subp. *Lactis* S40	*S. aurata*	-	-	+	+	-	++	-	-	-	*ent*P, *ent*B	-	-	-/-/-
*E. faecium* S38	*S. aurata*	-	-	+	+	-	++	-	-	-	-	-	-	-/-/-
*E. durans* S3	*M. merlangus*	-	-	+	+	-	+	-	-	+	*ent*P, *ent*B, *ent*1071A/B	*hyl*	E/*ermA*	-/-/-
*E. faecium* S10	*S. aurata*	+	-	++	+	++	++	-	-	++	*ent*AS48, *ent*1071A/B, *ent*B, *ent*L50A/B	-	-	-/-/-
*E. durans* S52	*S. aurata*	-	-	+	+	-	+	-	-	-	*ent*P	-	-	-/-/-
*E. faecium* S2	*S. aurata*	-	-	+	+	+	+	-	-	-	*ent*P, *ent*L50A/B	-	Lin/-	-/-/-
*E. faecium* S9	*M. merlangus*	-	-	+	+	-	-	-	-	-	*ent*1071A/B	-	Tet/ *tet*L + *tet*K	-/-/-
*E. faecalis* S44	*S. aurata*	-	-	-	+	-	+	-	-	+	*ent*AS48	-	Tet, E/ *tet*L + *tet*K, *ermA*	-/-/+ (α)
*E. faecium* S48	*S. aurata*	-	+	+	+	+	+	-	-	-	*ent*P	*hyl*	-	
*E. durans* S50	*S. aurata*	-	-	+	+	+	+	-	-	-	*ent*AS48, *ent*1071A/B	-	-	-/-/-
*E. faecium* S6	*M. merlangus*	-	-	+	++	+	-	-	-	++	*ent*B, *ent*1071A/B, *ent*L50A/B	-	-	-/-/-
*E. durans* S32	*M. merlangus*	-	+	++	++	+	+	-	-	-	*ent*1071A/B	-	-	-/-/-
*E. durans* S43	*S. aurata*	-	+	++	++	++	++	-	-	-	*ent*P	-	-	-/-/-
*E. faecium* S36	*S. aurata*	-	+	++	+	+	++	-	-	-	*ent*P	-	-	-/-/-
*E. durans* S45	*S. aurata*	-	+	+	++	+	++	-	-	-	*ent*P	-	-	-/-/-
*E. durans* S34	*M. merlangus*	-	-	+	+	+	-	-	-	-	-	-	Tet/ *tet*L + *tet*K	-/-/+ (α)
*E. faecium* S12	*M. merlangus*	-	+	+	+	+	+	-	-	-	*ent*P	-	-	-/-/-
*L. lactis* subp. *Lactis* S46	*S. aurata*	-	+	+	++	+	+	-	-	-	-	-	-	-/-/-
*E. faecium* S53	*S. aurata*	-	+	+	++	+	+	-	-	-	*ent*P	-	-	-/-/-
*E. faecalis* S51	*S. aurata*	-	-	++	++	++	++	-	-	+	*ent*P, *ent*AS48	-	E/*msrA*	-/+/-
*E. durans* S18	*M. merlangus*	-	-	+	+	++	++	-	-	+	*ent*P, *ent*1071A/B	-	E/*ermA*	-/-/-
*E. durans* S20	*M. merlangus*	+	-	+	+	-	++	-	-	-	-	-	Tet/ *tet*L + *tet*K	-/-/-
*E. faecium* S37	*S. aurata*	-	-	+	+	-	+	-	-	-	*ent*P	-	-	-/-/-
*E. faecium* S49	*S. aurata*	-	-	++	+	++	+	-	-	-	*ent*P	-	E/*mef*(A/E)	-/-/-
*E. durans* S25	*M. merlangus*	-	-	+	+	-	-	-	-	-	-	*hyl*	Tet/*tet*L + *tet*K	-/-/+ (α)
*E. durans* S35	*S. aurata*	-	-	++	++	-	++	-	-		*ent*P, *ent*X	-	-	-/-/+ (α)
*S. durans* S46	*S. aurata*	-	-	+	+	-	-	-	-	-	-	-	-	-/-/-
*E. faecium* S24	*M. merlangus*	-	-	+	+	-	+	-	-	-	*ent*P	-	Cip, E/-, *tet*L + *tet*K, *ermA*	-/+/-
*E. durans* S23	*M. merlangus*	-	-	+	+	-	+	-	-	-	*ent*P	-	-	-/-/-
*E. durans* S41	*S. aurata*	-	-	++	+	-	+	-	-	-	*ent*P	-	-	-/-/+ (α)
*E. faecium* S21	*M. merlangus*	-	-	+	+	-	+	-	-	-	*ent*P, *ent*1071A/B	-	-	-/-/-
*E. durans* S4	*M. merlangus*	+	-	++	-	-	++	-	-	++	*ent*P, *ent*B, *ent*1071A/B, *ent*L50A/B	-	-	-/-/-
*E. faecium* S7	*S. aurata*	+	-	++	-	-	++	-	-	++	*ent*B, *ent*1071A/B, *ent*L50A/B	-	-	-/-/-
*E. durans* S5	*S. aurata*	++	-	+	-	-	++	-	-	-	-	-	-	-/-/-
*E. faecium* S33	*M. merlangus*	-	+	+	+	-	+	-	-	-	-	-	-	-/-/-
*E. durans* S27	*M. merlangus*	++	-	++	-	-	++	-	-	-	*ent*B, *ent*L50A/B	-	-	-/-/-
*E. durans S28*	*M. merlangus*	+	+	++	-	-	++	-	-	-	-	-	Tet/*tet*L + *tet*K	-/-/+ (α)
*L. plantarum* S22	*M. merlangus*	-	-	++	-	-	+	-	-	-	-	-	-	-/-/-
*E. durans* S31	*M. merlangus*	++	-	++	+	-	++	-	-	-	*ent*P, *ent*B, *ent*L50A/B	*hyl*	-	-/+/-
*E. faecalis* S29	*M. merlangus*	-	-	-	++	-	-	-	-	-	*ent*AS48, *ent*1071A/B	-	Tet, E, St, Gen/*tet*L + *tet*K, *erm*A + *msr*A, -, *aac*(6′)-*aph*(2′’)	-/-/+ (α)
*E. faecalis* S8	*M. merlangus*	-	-	-	+	-	+	-	-	-	-	-	Tet/*tet*L + *tet*K	-/-/+ (α)
*L. lactis* subp. *Lactis* S26	*M. merlangus*	-	-	+	+	-	+	-	-	-	-	-	-	-/-/-

*S. aurata: Sparus aurata; M. merlangus: Merlangius merlangus; L. plantarum: Lactobacillus plantarum; L. lactis* subp. *lactis: Lactococcus lactis* spp. *lactis*; B.s: *Bacillus subtilis*; B.c: *Bacillus cereus*; S.a: *Staphylococcus aureus*; L.m: *Listeria monocytogenes* ATCC 43256; E.f: *Enterococcus faecalis* JH 2-2; P.l: *Paenibacillus larvae*; P.a: *Pseudomonas aeruginosa ATCC 27853*; V.a: *Vibrio alginolyticus*; Tet: tetracycline; E: erythromycin; Lin: linezolid; Cip: ciprofloxacin; St: streptomycin; Gen: gentamicin. * Scores were assigned based on the diameter of the inhibition halo: ++ = 5–9.9 mm, + = clear zone 1–4.99 mm, - = no zone.

## Data Availability

Data associated with this manuscript can be obtained from the corresponding author upon reasonable request.

## Supplementary Materials

The following supporting information can be downloaded at: https://www.mdpi.com/article/10.3390/life13091833/s1, Figure S1: Representative PCR gel for identification of *E. faecalis* and *E. faecium* species. M: 100 bp size marker. Lanes 1, 4, 5, and 6 correspond to isolates of *E. faecalis* (amplification of ddl_fc_ (941 bp)). Line 7: negative control (without DNA); Figure S2: Api 20 Strep of *E. faecium* isolate after incubation at 37 °C/24 h; Figure S3: Api 20 Strep of *E. faecalis* isolate after incubation at 37 °C/24 h.
