# Supplementary material for: Selection and Characterization of Bacteriocinogenic Lactic Acid Bacteria from the Intestine of Gilthead Seabream (Sparus aurata) and Whiting Fish (Merlangius merlangus): Promising Strains for Aquaculture Probiotic and Food Bio-Preservation
Source: Life (Basel). 2023 Aug 30;13(9):1833. doi: 10.3390/life13091833 (PMC10532712; doi:10.3390/life13091833)
Supplement: Supplementary file 1 [file life-13-01833-s001.zip › life-2506316-supplementary.pdf]

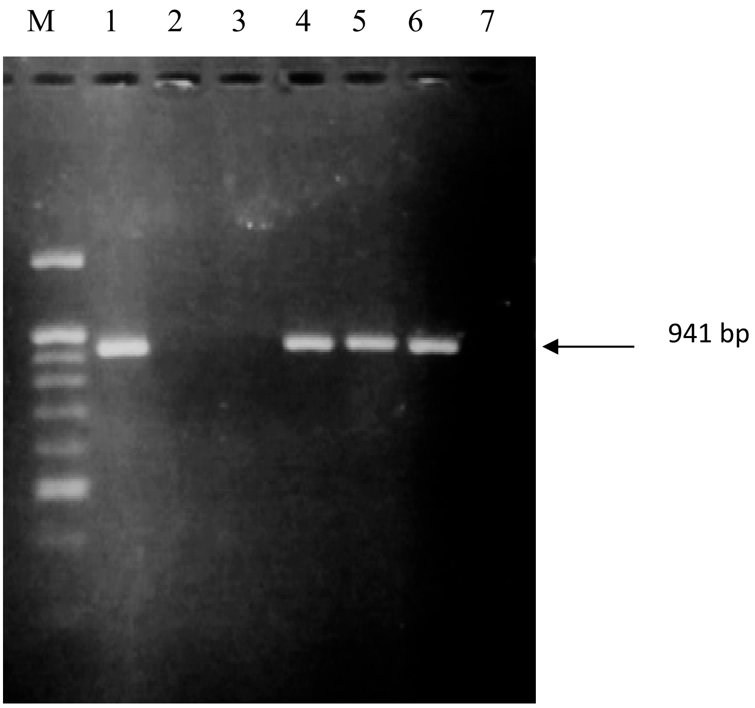

Figure S1. Representative PCR gel for identification of *E. faecalis* and *E. faecium* species. M: 100 bp size marker. Lanes 1, 4, 5, and 6 correspond to isolates of *E. faecalis* (amplification of ddl$_{fc}$ (941 bp)). Line 7: negative control (without DNA)

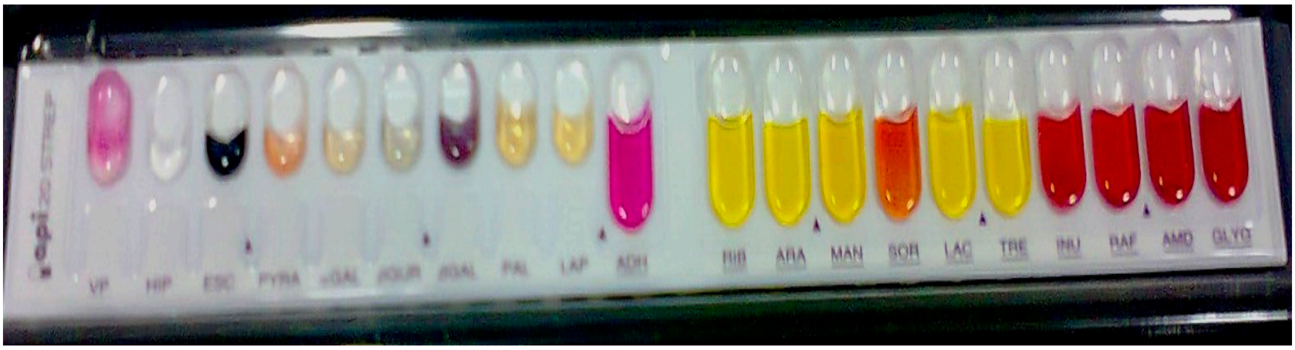

Figure S2. Api 20 Strep of *E. faecium* isolate after incubation at 37°C/24h

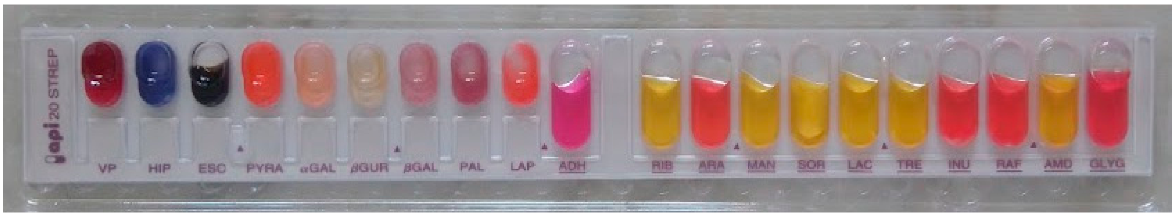

Figure S3. Api 20 Strep of *E. faecalis* isolate after incubation at 37°C/24h.
